# Abstracts and Items

**Published:** 1859-02

**Authors:** 


### ABSTRACTS AND ITEMS.

PREPARED BY THE JUNIOR EDITOR.

Dr. N. B. Benedict, in *N. O. Medical News*, reports a case of successful transfusion, in exhaustion from hemorrhage, in yellow fever. His object seems to have been to relieve the collapsed condition into which his patient had fallen, in consequence of the loss of blood. Is it not probable that transfusion may be used in these blood diseases, with great effect, to alter the character of the circulating mass, to avert deteriorating changes, and induce or promote healthy hematosis? If so, its range of usefulness would be very much expanded.

The Treatment of Burns, in the New York Hospital, consists in plastering the parts affected by the accident with the following paste, viz: Gum Arabic, ℥iii., gum tragacanth, ℥i., water, one pint, molasses ℥ii. It should be applied with a broad camel-hair pencil, once every two hours, until it forms a firm covering. This will require four or five applications. It should be allowed to remain on until it cracks and peels off, when the surface may be treated by the simplest means with success.—*Virginia Medical Journal.*

Dr. Joseph C. Hutchinson, of Brooklyn, and Dr. H. H. Toland, of San Francisco, record several cases of epistaxis hemorrhage, from wounds and other sources, treated in a very satisfactory manner, with a strong solution of persulphate of iron.

Dr. Kersey, of Indiana, in the *Nashville Journal of Med. and Surgery*, says that he gives large doses, say fifteen or twenty grains of act. of lead, with morphia and a little acetic acid, for strangulated hernia. He uses it in half-drachm doses per rectum for same purposes. He relates a case in which it seemed to exercise a powerful effect. He also uses muriated tincture of iron, undiluted, as an application to diphtheritic inflammation of the mucous membrane of the fauces and throat. It should be applied liberally, with a probang, about every eight hours.

THE FOLLOWING are the deductions of Dr. Vleminkx, of Brussels, on the subject of re-vaccination. He had 1660 cases upon which to base the conclusions. 1st. Re-vaccination of well-vaccinated individuals generally yields very few useful results. 2d. A person who has had small-pox should be more anxious to submit to re-vaccination than one who has been vaccinated. 3d. Re-vaccination succeeds the better the more distant the time of the operation is from the original vaccination, or from an attack of small-pox. 4th. Re-vaccination is useless up to the twenty-fifth year. 5th. From that period up to the thirty-fifth year, re-vaccination yields useful results, upon a certain number of individuals, but the number of the latter is exceedingly small; hence such re-vaccination should not be pressed upon people, though medical men should not altogether discountenance it. 6th. From the thirty-fifth year, re-vaccination becomes really preventive, and, therefore, necessary. 7th. Supposing the operation had failed once, it is no reason for not trying again, sometime afterward, as nothing proves that receptivity has not returned between the first and the subsequent operation. 8th. The re-vaccination of school-boys or girls is useless. 9th. Re-vaccination in armies organized like the Belgian (young soldiers) is also useless.—*Lancet*, from *Nashville Journal.*

CHLOROFORM IN DELIRIUM TREMENS.—Chloroform will sometimes allay the obstreperous restlessness of delirium tremens, and procure calm sleep when almost everything else fails us. We may cause our patients to inhale it with strong hopes of success.

DR. MARSHALL HALL'S new method of operating for stone in the bladder, is described as follows: He says, I propose to replace lithotomy and lithotrity by lithomy. 1st. Before the operation is performed, the patient should be made to drink copiously. 2d. A catheter, pierced with one hole only, should be introduced, and so placed as constantly to leave in the bladder such a quantity of urine as seems desirable. 3d. A canula, supplied with a sharp point, should be thrust into the bladder above the os pubis and below the peritonæum. 4th. The point (or stillet.—Ed.) is to be withdrawn and the canula left, closed by a little stop-cock, until a fistula, thoroughly surrounded by lymph, be well established. 5th. This fistula should then be properly dilated until the calculus can be extracted. 6th. The fistula is afterward allowed gradually to contract, on an india-rubber tube, distended with air to a proper size, which is gradually to diminish in calibre by the air being made to escape. In the whole course of this operation no artery is divided; no blood flows; and the nervous system experiences no shock. A measure new in surgery is thus employed, viz: dilatation instead of division of textures. This measure, when well conducted, is not dangerous to the patient, and offers no difficulty to the operation. It may be also applied to other circumstances, such as hydropericardium, empyema, and to replace tracheotomy.—*London Lancet.*

Dr. Richard Bright, who has given his name to the peculiar disease of the kidney known as Bright's disease, is dead. Drs. Lever and William Cruikshanks, both of whose names are quite familiar to American students, have likewise recently departed this life.—*London Lancet.*

